# Use of First-Line Oral Analgesics during and after COVID-19: Results from a Survey on a Sample of Italian 696 COVID-19 Survivors with Post-Acute Symptoms

**DOI:** 10.3390/jcm12082992

**Published:** 2023-04-20

**Authors:** Vincenzo Galluzzo, Maria Beatrice Zazzara, Francesca Ciciarello, Matteo Tosato, Alessandra Bizzarro, Annamaria Paglionico, Valentina Varriano, Elisa Gremese, Riccardo Calvani, Francesco Landi

**Affiliations:** 1 Department of Geriatrics, Orthopedics and Rheumatology, Fondazione Policlinico Universitario “Agostino Gemelli” IRCCS, 00168 Rome, Italy; 2 Department of Geriatrics and Orthopedics, Università Cattolica del Sacro Cuore, 00168 Rome, Italy

**Keywords:** arthralgia, joint pain, myalgia, headache, COVID-19, long COVID, acetaminophen, ibuprofen, NSAIDs, geriatrics

## Abstract

**Highlights:**

**What are the main findings of the study?**

- The most-used pain medication during COVID-19 is acetaminophen.
- The most common analgesics used in post-COVID-19 are acetaminophen, ibuprofen or other NSAID, while older subjects prefer acetaminophen.
- The frequency of pain medication is often several times a week.

**What is the implication of the main findings?**

- The need to continue analgesic therapy after COVID-19 is associated with persistent arthralgia and myalgia.
- Most patients report an improvement in pain perception after taking analgesic therapy.

**Abstract:**

Background—Analgesics could be used to manage painful symptoms during and after COVID-19. Materials and methods—Persistence of painful symptoms was assessed during and after COVID-19 in a sample of patients admitted to a post-acute COVID-19 outpatient service in Rome, Italy. Data on type and frequency of use of first-line analgesics were collected. Pain severity was evaluated with a numeric rating scale (NRS) from 0 to 10. Results—Mean age of 696 participants was 57.1 ± 20.3 years and 61.7% were women. During COVID-19, the most prevalent symptoms were fever, fatigue, arthralgia, myalgia and headache. Acetaminophen was used by 40% of the sample. Only 6.7% needed to continue analgesic therapy after COVID-19. Frequent causes of analgesics consumption were persistent arthralgia and myalgia. The most common analgesics used amongst those who continued taking analgesics in the post-acute phase of COVID-19 were the following: acetaminophen (31%), ibuprofen (31%) and other non-steroidal anti-inflammatory drug (NSAID) (29.5%); in older subjects the most common analgesic used was acetaminophen (54%). Most of the subjects in this group said there was an improvement in pain perception after taking analgesic therapy (84%). Conclusions—Use of analgesics in the post-acute COVID-19 is common in subjects with persistent arthralgia and myalgia, and common analgesics were acetaminophen and ibuprofen. Further research on the safety and efficacy of those medications in COVID-19 is warranted.

## 1. Introduction 

Acute COVID-19 is mainly characterized by upper airway symptoms [1], and can lead to acute respiratory distress syndrome (ARDS) and multiple organ failure that require immediate hospital admission [2,3]. Clinical consequences of acute Sars-CoV2 infection can vary from paucisymptomatic to moderate-severe symptoms [4], and long-lasting sequalae might involve different anatomical districts [5]. Medium- to long-term symptoms that arise within 3 months after the infection configure a clinical condition called long Covid [6]. In the acute and post-acute phase of COVID-19, painful symptoms are primarily associated with arthralgia, myalgia, sore throat, chest pain and headache [7,8]. Molecular mechanisms that play a role the onset and persistence of these symptoms are still under investigation [9,10,11,12]. Frequently, chest pain is not directly associated with a cardiac origin [13], while headaches are often accompanied by sleep disorders or difficulty concentrating [14]. These symptoms are present even in mild COVID-19, and common first-line analgesics might be useful in the short-to-medium-term treatment of pain during acute and post-acute COVID-19 [15]. Although non-steroidal anti-inflammatory drugs (NSAIDs) do not reduce the risk of the worsening of COVID-19 symptoms, the use of these medications might represent an alternative strategy to acetaminophen in the treatment of fever or pain during COVID-19 [16]. Research has demonstrated that both ibuprofen and acetaminophen show a similar efficacy on pain in different forms of headache [17,18]. NSAIDs are also more effective than acetaminophen on pain relief related to osteoarthritis [19,20], albeit they are associated with greater gastrointestinal side effects [21]. Moreover, NSAIDs represent a common option in the management of chronic low back pain [22]. In all forms of acute pain, single doses of ibuprofen produce good pain relief in more people than acetaminophen alone [19]. Considering that painful symptoms might persist for several weeks during the post-acute phase, the intermittent use of first-line analgesics could be common in long COVID patients.

With this study, we aim to assess type and frequency of first-line oral analgesics’ consumption during and after COVID-19 in a sample of individuals who are still suffering from post-acute symptoms.

## 2. Materials and Methods

The Gemelli Against COVID-19 Post-Acute Care (GAC19-PAC) project is an ongoing initiative developed by the Department of Geriatrics, Neuroscience and Orthopedics of the Catholic University of the Sacred Heart (Rome, Italy) that started in April 2020 to investigate the long-term consequences of COVID-19 and its impact on overall health and quality of life. Details on the post-acute outpatient service of Fondazione Policlinico Gemelli (Rome, IT) and patient evaluation are reported elsewhere [5].

We conducted a descriptive study on individuals aged > 18 years old recovered from COVID-19 and admitted to the post-COVID-19 outpatient service between 1 March 2022 and 30 November 2022. Informed consent was obtained by all the patients upon admission to the clinic. Symptoms that arose during or immediately after SARS-CoV-2 infection and persisted at the time of the evaluation have been included as sequalae of COVID-19 [23,24,25,26].

All patients admitted to our post-COVID-19 outpatient clinic were offered a comprehensive medical assessment. Medical history, current medications, lifestyle habits, marital status, cohabitation status and occupational status were collected in a structured electronic database.

The presence and severity of principal painful symptoms (arthralgia, myalgia, headache, chest pain) were assessed during the visit and concerned both the acute phase and the post-acute phase. For pain severity, a numeric rating scale (NRS) from 0 to 10 was considered (0 corresponds to absence of pain; 10 indicates pain at the maximum level). The use of analgesics was also assessed and a list of oral analgesics was considered as follows: acetaminophen, ibuprofen, other NSAIDs, combination of acetaminophen and ibuprofen, combination of acetaminophen and other analgesic (non-ibuprofen, non-other NSAIDs) [15]. The frequency of use of analgesic therapy was further assessed in those participants who declared to have continued using analgesics during the post-acute COVID-19. The frequency of use was categorized as follows: daily, for several days per week, one week per month, once a week, once a month. Treatment with steroids was assessed only during acute infection. 

In addition, information regarding pre- and post-COVID-19 self-rated health was collected using a visual analogic scale (VAS), where 0 indicates worst perceived health status and 100 shows best perceived health status. Information on physical activity levels prior to the acute SARS-CoV-2 infection and at the time of the evaluation was also collected. Regular participation in physical activity was operationalized as the engagement in aerobic physical activity, whether or not it was associated with resistance training, for a minimum of 150 min per week in the last 3 months.

For data presentation purposes, the sample was divided in two groups, according to the need to continue analgesic therapy in the post-acute COVID-19 (group YES: subjects that continued analgesics’ consumption after COVID-19; group NO: subjects who do not consume analgesics after COVID-19). The decision to continue taking analgesics after COVID-19 was made autonomously or under the advice of the general practitioner.

Descriptive statistics were used to describe demographic and key clinical characteristics of the study population: overall and according to analgesic use during the post-acute phase (group YES and group NO). Continuous variables are presented as mean ± standard deviation (SD), and categorical variables are presented as frequencies by absolute value and percentages (%). All data were collected using REDCap, a software that enables structured electronic data collection. Statistical analyses were performed using R statistical environment (version 4.0).

## 3. Results

Among patients referred to the post-COVID-19 outpatient service of Policlinico Gemelli in Rome between March and November 2022, a sample of 696 individuals still suffering from post-acute symptoms was identified. The YES group represented 6.3% of the total sample. Characteristics of the study population according to the two groups of interest are shown in Table 1. The average amount of days from COVID-19 diagnosis to admission to the outpatient service was 340.86 days. The mean age of the sample was 57.1 ± 20.3, and 430 (61.7%) were females. In our sample, 340 (48.9%) were married, 437 (62.8%) had a full- or part-time occupation and 226 (32.5%) lived with their family during the acute phase of the infection. 

In the YES group, 36% were hospitalized and more than 20% required oxygen therapy during the acute phase. Use of analgesic medications in the post-acute COVID-19 was more common in single individuals, who lived alone or were retired. In addition, the prevalence of chronic conditions (cardiovascular, metabolic, autoimmune, gastrointestinal/hepatic, psychiatric, genitourinary, neurological and musculoskeletal diseases) was higher in the YES group, while the prevalence of diabetic individuals or those with cancer was less common or absent. The prevalence of smokers was higher in the NO group. 

As reported in Table 2, the most prevalent symptoms during COVID-19 were fever, fatigue, arthralgia, myalgia and headache. The NO group manifested sore throat more frequently in the post-acute phase, although it was not associated with the need to take analgesic therapy. Approximately 40% of individuals in the two groups have taken steroids and acetaminophen during COVID-19, followed by ibuprofen (22%). The most common analgesics used in the post COVID-19 were the following: acetaminophen (31%), ibuprofen (31%), other NSAID (29.5%) and a combination of acetaminophen with other analgesic (11.4%). In the post COVID-19 phase, the frequency of acetaminophen’s consumption was once or several times a week; for ibuprofen, it was several times a week, followed by daily or once a week. Individuals in the NO group took supplements more frequently in the post-acute phase. Although the median severity of pain did not change between the two phases, the majority of individuals in the YES group referred an improvement in pain perception after taking analgesic therapy (84%). 

Regarding solely participants aged over 65 years old (Table 3 and Table 4), steroids were taken by nearly 50% of subjects during the acute phase of COVID-19. The consumption of acetaminophen and ibuprofen was frequent during acute COVID-19, but lower than the total sample. A large number of participants aged 65 years old in the YES group manifested post-COVID-19 arthralgia and myalgia. In contrast with the total sample, acetaminophen was the most commonly used analgesic (54%). Alternatively, the most-used analgesics were a combination of acetaminophen and other analgesic (27%) or ibuprofen (18%). The frequency of acetaminophen’s consumption was several times a week. 

## 4. Discussion

We conducted a descriptive study to investigate the use of first-line oral analgesics in a sample of individuals affected by long COVID. We noticed that a high proportion of the study population did not declare to use any analgesics in the post-acute COVID-19. While we found a higher proportion of individuals who had been hospitalized for COVID-19 in the group of participants who continued analgesics during the post-acute phase, our data show that older participants, who are usually more severely affected by COVID-19, referred to have discontinued analgesics in this phase. Most likely the perception of pain in this category of patients, and consequently the level of self-rated health, is different from younger individuals and undermined by older subjects who might tend to be more accustomed to musculoskeletal symptoms [27]. Furthermore, a recent meta-analysis has shown that the frequency of myalgia and arthralgia in acute COVID-19 is similar between hospitalized and non-hospitalized patients, although the incidence of these symptoms tends to be higher in hospitalized subjects up 60 to 90 days after first viral detection [28]. As seen in those who did not continue to take analgesics in the post-acute phase, we cannot undermine the role of supplements that are often used in long-term management of persistent post-COVID-19 symptoms [11,29,30,31,32].

Among those who continued using analgesics in the post-acute COVID-19, we found a high percentage of women, while the use of analgesics was less frequent amongst subjects with diabetes or cancer and smokers. Women, in fact, tend to suffer from long COVID symptoms more than men [33,34]. On the other hand, pain perception in diabetes tends to be impaired, especially in patients who develop painful and non-painful diabetic neuropathy [35], and several molecular mechanisms co-participate in the deterioration of small nerve fiber function that alter the pain threshold in diabetic subjects [35], while participants with cancer might have already been treated with other pain medications such as opioids to cope with cancer pain, being that first-line analgesics were insufficient to manage it [36,37]. Finally, evidence has demonstrated that individuals manifesting pain are more reluctant to quit smoking [38]. Indeed, the urge to smoke for pain relief is strictly associated with pain severity [39]. 

Concerning the type of oral first-line analgesics, we found that one third of our sample consumed acetaminophen and one of five participants used ibuprofen during acute COVID-19. Although acetaminophen and NSAIDs are not indicated in the treatment of acute COVID-19 by international guidelines, these medications represent effective drugs to control fever and sore throat [40,41]. Current literature reports only one randomized trial that emphasizes the role of naproxen in the improvement of acute COVID-19 symptoms such as cough or shortness of breath [42]. Nevertheless, prior treatment with NSAIDs is associated with a decreased risk of severe COVID-19 and death [43]. The frequency of steroids assumption in our sample was higher among older subjects, probably in relation to disease severity and hospitalization rate [44]. Amongst the group that continued using analgesics in the post-acute phase of COVID-19, we found that the most common-used drugs were acetaminophen and NSAIDs. Half of the individuals that consumed NSAIDs choose ibuprofen, which is one the most effective non-opioid oral pain medications and is generally appropriated for the management of acute pain [45]. First-line analgesics, particularly NSAIDs, are effective in controlling pain severity related to osteoarthritis [46]. Several studies have demonstrated the superiority of ibuprofen to acetaminophen for the management of acute pain, migraine and osteoarthritis [19]. A combination of acetaminophen and ibuprofen could represent a valid option for pain control [19], although the long-term use of ibuprofen is associated with increased risk of peptic ulcer, chronic kidney injury and heart failure [47,48,49]. Moreover, the use of NSAIDs during acute COVID-19 might augment the risk of stroke [43]. When addressing older adults, acetaminophen or a combination with acetaminophen and other analgesic, which are associated with less severe side effects, should be preferred [50]. Finally, during the post-acute phase of COVID-19, those who continued using analgesics said that they consumed them frequently in response to pain because of the beneficial effect of the therapy and temporary reduction in pain severity.

This study has several limitations. The descriptive nature of this study does not permit the capture of significant changes in pain perception over time, nor does it assess potential correlation or derive any conclusion on association of presence of pain, Long COVID and the use of different first-line analgesics. Furthermore, the evaluation of pain severity with validated scales was not available. We also lack information about the use of analgesic medications before COVID-19. Finally, although our sample included predominantly unselected patients with painful and non-painful post-COVID-19 symptoms attending an outpatient service, a selection bias cannot be ruled out.

## 5. Conclusions

We described the use of oral first-line analgesics during COVID-19 and after the infection in a sample of COVID-19 survivors with post-acute symptoms. The use of analgesics is more common in those manifest post-COVID-19 arthralgia and myalgia. Acetaminophen and ibuprofen are the most-used pain medications during COVID-19. Among oral first-line analgesics, post-acute COVID-19 patients choose acetaminophen, ibuprofen or another NSAIDs, while older subjects prefer to consume acetaminophen or a combination of acetaminophen and another analgesic. The frequency of analgesics’ consumption is often “several times a week”. Most individuals refer to an improvement in pain perception after starting analgesic therapy. However, considering that post-acute COVID-19 symptoms can persist for several months, self-management of pain should not be encouraged and all COVID-19 patients with long-term effects should be referred to specialists or assessed by a multidisciplinary team to tailor a patient-center care plan and avoid potential adverse events or risk of addiction. Future studies are warranted to assess which medications might be appropriated to obtain pain relief following Sars-CoV-2 infection and if the consumption of first-line analgesics might be effective and safe in acute and post-acute COVID-19 management.

## Figures and Tables

**Table 1:** Characteristics of study population according to the need to continue analgesic therapy in the post-acute COVID-19 (YES: those who continued taking analgesics in the post-acute phase; NO: those who did not).

	Total Sample (n: 696)	Yes (n: 44)	No (n: 652)
General and Clinical Characteristics			
Female (%)	430 (61.7%)	36 (81%)	394 (60%)
Age (mean ± ds)	57.1 (20.3)	51.5 (15.9)	57.5 (20.5)
Age (median) [range]	43 [22–89]	49 [22–89]	43 [18–72]
Body Mass Index (BMI) (m^2^/kg) (mean ± ds)	25.9 (5.25)	27.3 (6.0)	25.8 (5.25)
Marital status			
Married	340 (48.9%)	22 (50%)	318 (48.8%)
Divorced	82 (11.8%)	2 (4.45%)	80 (12.3%)
In other type of relationship	8 (0.9%)	6 (13.6%)	2 (0.3%)
Separated	26 (3.7%)	2 (4.45%)	24 (3.7%)
Widowed	23 (3.3%)	1 (2.27%)	22 (3.4%)
Single	41 (5.9%)	11 (25%)	30 (4.6%)
Cohabitation status			
Alone	96 (13.8%)	9 (20.45%)	87 (13.3%)
With only the partner	170 (24.4%)	13 (29.5%)	157 (24%)
With only the sons	39 (5.6%)	3 (6.8%)	36 (5.5%)
With partner and sons	226 (32.5%)	15 (30.1%)	211 (32%)
With parents	48 (6.9%)	2 (4.45%)	46 (7%)
With brothers	2 (0.3%)	0	2 (0.3%)
With other relatives	9 (1.3%)	2 (4.45%)	7 (1%)
Occupational status			
Employed	437 (62.8%)	28 (63.6%)	409 (62.7%)
Unemployed	73 (10.5%)	5 (11.36%)	68 (10.4%)
Retired	95 (13.6%)	10 (22.7%)	85 (13%)
Other	16 (2.3%)	1 (2.27%)	15 (2.3%)
Comorbidities			
Hypertension	139 (20%)	9 (20.45%)	121 (18.6%)
Atrial Fibrillation	19 (2.7%)	3 (6.8%)	16 (2.5%)
Other CV Diseases	52 (7.5%)	5 (11.36%)	47 (7.2%)
Diabetes	57 (8.2%)	1 (2.27%)	56 (8.6%)
Other Metabolic Disorders	41 (5.9%)	4 (9.1%)	37 (5.7%)
Thyroid Diseases	177 (25.4%)	15 (30.1%)	162 (24.8%)
COPD/asthma/OSAS	35 (5%)	3 (6.8%)	32 (4.9%)
Autoimmune Diseases	59 (8.5%)	5 (11.36%)	54 (8.3%)
Liver and Gastrointestinal Diseases	50 (7.2%)	12 (27.27%)	38 (5.8%)
Renal failure	13 (1.9%)	1 (2.27%)	12 (1.8%)
Psychiatric Diseases	21 (3%)	5 (11.36%)	16 (2.5%)
Genitourinary Diseases	32 (4.6%)	7 (15.9%)	25 (3.8%)
Nervous System Diseases	33 (4.7%)	4 (9.1%)	29 (4.4%)
Musculoskeletal Diseases	13 (1.9%)	6 (13.6%)	7 (1%)
Cancer	12 (1.7%)	0	12 (1.8%)
Tobacco Use			
Never smoke	443 (63.6%)	25 (56%)	418 (64%)
Former smoker	166 (23.9%)	13 (29.5%)	153 (23.5%)
Smoker	78 (11.2%)	3 (6.8%)	75 (11.5%)
Therapies and Allergies			
Number of daily medications (median) [range]	1 [0–13]	1 [0–11]	1 [0–13]
Subjects with allergies	224 (32.2%)	16 (36%)	208 (32%)
Subjects with allergies to medications	97 (14%)	9 (20.45%)	88 (13.5%)
COVID-19			
Average days from COVID-19 diagnosis (mean ± ds)	340.86 (197.36)	324.27 (225.97)	341.98 (197.92)
Hospital admission	154 (22%)	16 (36%)	136 (20.8%)
ICU admission	22 (3.2%)	3 (6.8%)	19 (2.9%)
O2 support	88 (12.6%)	9 (20.45%)	79 (12%)
NIV	37 (5.3%)	4 (9.1%)	33 (5%)
Invasive Ventilation	12 (1.7%)	2 (4.45%)	10 (1.5%)
Sars-CoV-2 Vaccination *	585 (84%)	37 (84%)	548 (89.6%)

* at least one dose. CV: Cardiovascular. COPD: Chronic obstructive pulmonary disease. OSAS: Obstructive sleep apnea syndrome. ICU: Intensive care unit. NIV: Non-invasive ventilation.

**Table 2:** Symptoms during and after COVID-19 and utilization of analgesic therapies in the groups of interest (YES: those who continued taking analgesics in the post-acute phase; NO: those who did not).

	Total Sample (n: 696)		Yes (n: 44)		No (n: 652)	
	*During COVID*	*After COVID*	*During COVID*	*After COVID*	*During COVID*	*After COVID*
Symptoms						
Arthralgia	305 (44%)	151 (22%)	26 (59%)	24 (54%)	279 (43%)	127 (20%)
➢ Severity (Median) (Range)	8 (2–10)	8 (3–10)	8 (6–10)	8 (3–10)	8 (2–10)	8 (3–10)
Myalgia	337 (48%)	174 (25%)	32 (72%)	29 (65%)	305 (47%)	145 (22%)
➢ Severity (Median) (Range)	8 (0–10)	7 (3–10)	8 (5–10)	8 (3–10)	8 (0–10)	7 (3–10)
Headache	323 (46%)	140 (20%)	31 (70%)	18 (40%)	292 (45%)	122 (19%)
➢ Severity (Median) (Range)	7 (2–10)	7 (2–10)	8 (2–10)	7.5 (3–10)	7 (2–10)	7 (2–10)
Chest pain	177 (25%)	72 (10%)	17 (38%)	4 (9%)	160 (25%)	68 (10%)
➢ Severity (Median) 8(Range)	7 (2–10)	7 (2–10)	7.5 (5–10)	7 (7–9)	7 (2–10)	7 (2–10)
Sore throat	242 (35%)	18 (2.6%)	15 (34%)	0	227 (35%)	18 (3%)
Fever	459 (66%)	9 (1.3%)	37 (84%)	1 (2.27%)	422 (68%)	8 (1%)
Fatigue	490 (70%)	430 (62%)	41 (93%)	33 (75%)	449 (69%)	397 (61%)
GORD	82 (12%)	73 (11%)	14 (31%)	3 (6.8%)	68 (10%)	70 (11%)
Cough	354 (51%)	56 (8%)	29 (66%)	3 (6.8%)	325 (50%)	53 (8%)
Dizziness	118 (17%)	50 (7%)	17 (38%)	5 (11.4%)	101 (15%)	45 (7%)
Dyspnea	303 (44%)	281 (40%)	24 (54%)	25 (56%)	279 (43%)	256 (39%)
Brain fog	119 (17%)	163 (23%)	17 (38%)	15 (34%)	102 (16%)	148 (23%)
Tingles	85 (12%)	85 (12%)	14 (31%)	15 (34%)	71 (11%)	70 (11%)
Pharmacological therapies						
Steroids	293 (42%)		21 (48%)		272 (42%)	
Acetaminophen	275 (40%)	14 (2%)	19 (44%)	14 (31%)	256 (39%)	
➢ Frequency				Daily 1 (7%) Several times/wk 6 (43%) Once a wk 5 (36%) 1 wk per month 1 (7%) Once a month 1 (7%)		
Acetaminophen and Ibuprofen	21 (3%)	1 (0.14%)	18 (40%)	1 (2.27%)	3 (0.5%)	
➢ Frequency				Daily 1(100%)		
Ibuprofen	155 (22%)	14 (2%)	18 (40%)	14 (31%)	137 (21%)	
➢ Frequency				Daily 3 (21%) Several times/wk 7 (50%) Once a wk 4 (29%)		
Other NSAIDs	51 (7.3%)	13 (1.9%)	2 (4.45%)	13 (29.5%)	49 (7.5%)	
➢ Frequency				Daily 2 (14%) Several times/wk 9 (70%) Once a wk 1 (8%) Once a month 1 (8%)		
Acetaminophen and other analgesic	4 (0.6%)	5 (0.72%)	1 (2.27%)	5 (11.36%)	3 (0.5%)	
➢ Frequency				Daily 2 (40%) Several times/wk 3 (60%)		
Have you noticed an improvement in pain perception as a result of taking analgesic therapy?				Yes 37 (84%) No 7 (16%)		
Supplements	160 (23%)	222 (32%)	6 (13.6%)	7 (15.9%)	154 (24%)	215 (33%)
Homeopathic products	12 (1.7%)	12 (1.7%)	0	0	12 (1.8%)	12 (1.8%)
Herbal products	10 (1.4%)	13 (1.9%)	0	0	10 (1.5%)	13 (2%)
Other Characteristics						
	*Before COVID*	*After COVID*	*Before COVID*	*After COVID*	*Before COVID*	*After COVID*
Regular physical activity	354 (51%)	247 (35%)	21 (47.7%)	11 (25%)	333 (51%)	236 (36%)
Self-rated health (Median) [Range]	82 [20–100]	65 [0–100]	80 [30–97]	50 [0–80]	83 [20–100]	70 [0–100]

GORD: Gastro-esophageal reflux disease. NSAIDs: Non-steroidal anti-inflammatory drugs.

**Table 3:** Characteristics of older subjects according to the need to continue analgesic therapy in the post-acute COVID-19 (YES: those who continued taking analgesics in the post-acute phase; NO: those who did not).

	Older Yes (n: 11)	Older No (n: 109)
General and Clinical Characteristics		
Female (%)	8 (72%)	62 (57%)
Age (mean ± ds)	73.4 (8.05)	72.4 (6.27)
Age (median) [range]	73 [65–89]	71 [65–96]
Body Mass Index (BMI) (m^2^/kg) (mean ± ds)	27.9 (5.5)	27.1 (0.82)
Marital status		
Married	8 (73%)	72 (67%)
Separated	1 (9%)	11 (10%)
Widowed	1 (9%)	21 (19%)
In other type of relationship	1 (9%)	0
Single	0	5 (4%)
Cohabitation status		
Alone	1 (9%)	17 (16%)
With only the partner	8 (73%)	71 (65%)
With only the sons	1 (9%)	8 (7%)
With partner and sons	1 (9%)	13 (12%)
Comorbidities		
Hypertension	6 (54%)	46 (42%)
Atrial Fibrallation	3 (27%)	12 (11%)
Other CV Diseases	0	12 (11%)
Diabetes	1 (10%)	16 (15%)
Thyroid Diseases	6 (54%)	22 (20%)
COPD/Asthma/OSAS	2 (18%)	7 (6%)
Autoimmune Diseases	2 (18%)	2 (2%)
Liver and Gastrointestinal Diseases	1 (10%)	1 (1%)
Renal failure	1 (10%)	9 (8%)
Cancer	0	1 (1%)
Therapies		
Number of daily medications (median) [range]	3 [1–10]	3 [0–10]
COVID-19		
Hospital admission	6 (60%)	41 (51%)
ICU admission	1 (10%)	7 (6%)
O_2_ support	3 (27%)	33 (30%)
NIV	2 (18%)	7 (6%)
Invasive Ventilation	1 (10%)	1 (1%)

CV: cardiovascular. COPD: Chronic obstructive pulmonary disease. OSAS: Obstructive sleep apnea syndrome. ICU: Intensive care unit. NIV: Non-invasive ventilation.

**Table 4:** Symptoms during and after COVID-19 and utilization of analgesic therapies in older subjects (YES: those who continued taking analgesics in the post-acute phase; NO: those who did not).

	Older Yes (n: 10)		Older No (n: 109)	
	*During COVID*	*After COVID*	*During COVID*	*After COVID*
Symptoms				
Arthralgia	8 (72%)	10 (91%)	41 (38%)	26 (24%)
➢ Severity (Median) [Range]	9 [6–10]	8 [7–10]	8 [3–10]	8 [4–9]
Myalgia	8 (72%)	10 (91%)	39 (36%)	25 (23%)
➢ Severity (Median) [Range]	9 [6–10]	8 [7–10]	8 [3–10]	7 [4–9]
Headache	5 (45%)	2 (18%)	27 (25%)	14 (13%)
➢ Severity (Median) [Range]	7 [3–9]	5 [3–7]	8 [3–10]	7 [3–9]
Chest pain	3 (27%)	0	17 (16%)	7 (6%)
➢ Severity (Median) [Range]	6.5 [6–7]	0	8 [3–9]	4 [4–9]
Sore throat	3 (27%)	0	22 (20%)	0
Fever	9 (82%)	1 (10%)	81 (74%)	0
Fatigue	10 (91%)	6 (54%)	76 (70%)	69 (63%)
GORD	2 (18%)	1 (10%)	6 (6%)	7 (6%)
Cough	10 (91%)	2 (18%)	57 (52%)	8 (7%)
Dizziness	3 (27%)	2 (18%)	12 (11%)	8 (7%)
Dyspnea	6 (54%)	5 (45%)	47 (42%)	47 (42%)
Brain fog	3 (27%)	3 (27%)	12 (11%)	22 (20%)
Tingles	3 (27%)	4 (36%)	9 (8%)	11 (10%)
Pharmacological therapies				
Steroids	5 (45%)		52 (48%)	
Acetaminophen	3 (27%)	6 (54%)	37 (34%)	
➢ Frequency		Several times/wk 4 (66%) Once a wk 1 (17%) 1 wk per month 1 (17%)		
Acetaminophen and Ibuprofen	0	1 (10%)	0	
➢ Frequency		Daily 1 (100%)		
Ibuprofen	2 (18%)	2 (18%)	16 (15%)	
➢ Frequency		Daily 1 (50%) Several times/wk 1 (50%)		
Other NSAIDs	1 (10%)	1 (10%)	8 (7%)	
➢ Frequency		Daily 1(100%)		
Acetaminophen and other analgesic	0	3 (27%)	1 (1%)	
➢ Frequency		Daily 1 (33%) Several times/wk 2 (67%)		
Have you noticed an improvement in pain perception as a result of taking analgesic therapy?		Yes 9 (82%) No 2 (18%)		
Supplements	1 (10%)	1 (10%)	26 (27%)	34 (31%)
Homeopathic products	0	0	1 (1%)	1 (1%)
Herbal products	0	0	2 (2%)	1 (1%)
Other Characteristics				
	*Before COVID*	*After COVID*	*During COVID*	*After COVID*
Regular physical activity	7 (64%)	4 (36%)	48 (46%)	32 (30%)
Self-rated health (Median) [Range]	72 [63–93]	50 [27–74]	80 [50–100]	65 [23–90]

## Data Availability

All the data and material are available.
